# Influence of different therapy strategies in patients with myelodysplastic neoplasms (MDS) on overall survival with regard to different decades: data from the German MDS registry

**DOI:** 10.1007/s00277-026-06736-4

**Published:** 2026-04-13

**Authors:** Jonas Welsch, Felicitas Schulz, Annika Kasprzak, Carlo Aul, Katharina S. Götze, Arnd Nusch, Jörg Lipke, Stefani Parmentier, Corinna Strupp, Kathrin Nachtkamp, Guido Kobbe, Sascha Dietrich, Norbert Gattermann, Ulrich Germing

**Affiliations:** 1 Department of Hematology, Oncology and Clinical Immunology, University Hospital Duesseldorf, Duesseldorf, Germany; 2 Department of Medicine III, Hematology and Medical Oncology, Technical University of Munich, TUM Universitätsklinikum/Klinikum rechts der Isar, Munich, Germany; 3 Department of Medicine II, St. Johannes Hospital, Duisburg, Germany; 4 Practice for Hematology and Internal Oncology, Velbert, Germany; 5 Practice for Hematology and Internal Oncology, Dortmund, Germany; 6 Tumorzentrum Claraspital, Claraspital Basel, Basel, Switzerland

**Keywords:** AML, BSC, MDS, Prognosis, Survival, Treatment

## Abstract

Over the history of myelodysplastic neoplasms (MDS), an increasing number of treatment modalities have become available. It is therefore important to assess whether these developments have translated into improved overall survival (OS) for patients. We analyzed data from 1,147 patients in the German MDS registry and observed a progressive improvement in median survival across three diagnostic periods: until 2000 (20 months), 2001–2010 (31 months), and after 2011 (57 months). Patients treated with allogeneic stem cell transplantation (SCT) had the best prognosis with a median survival of 108 months. Over time, both the proportion of patients undergoing allogeneic SCT (6.3% vs. 22.1% vs. 40.0%) and the median age at transplantation (36.5 vs. 52 vs. 61 years) increased. Notably, OS also improved among patients treated with best supportive care (BSC) alone (17 vs. 31 vs. 64 months; *p* < 0.001). Multivariate analysis demonstrated that the period of first diagnosis had an independent positive effect on survival. The underlying reasons for this improvement remain unclear, but a multifactorial origin—including improved supportive measures and greater eligibility for allogeneic SCT—must be considered.

## Introduction

Myelodysplastic neoplasms (MDS) are a heterogeneous group of clonal disorders of the bone marrow characterized by an ineffective hematopoiesis. This leads to dysplasia of hematopoietic cells which results in bone marrow failure. Common clinical manifestations include anemia, increased susceptibility to infections and bleeding complications [1]. In addition patients have an increased risk of progression to acute myeloid leukemia (AML). MDS were first described in 1982 by the French-American-British (FAB) group [2]. The current classification system of the World Health Organization (WHO) [3] is based on cytomorphological criteria, bone marrow blast count and molecular as well as cytogenic findings.

Since the initial classification of MDS in 1982, therapeutic approaches have evolved substantially. At first, no disease-modifying treatments were available, and patients were managed primarily with supportive transfusions. In the 1990 s, hematopoietic growth factors such as erythropoietin and granulocyte colony-stimulating factor (G-CSF), as well as iron chelation therapy, began to be used more systematically. Induction chemotherapy became more prevalent before the turn of the millennium. After 2000, hypomethylating agents, particularly 5-azacytidine, were increasingly implemented.

Currently, immunomodulators and growth factors are preferred for patients with low-risk MDS, while hypomethylating agents remain the standard treatment in high-risk cases. Allogeneic hematopoietic stem cell transplantation (allogeneic SCT) continues to be the only curative option. It is typically reserved for high-risk patients with adequate performance status and for selected low-risk individuals with high-risk cytogenetics/molecular genetic findings or significant cytopenias [4].

Given the continuous development of therapeutic strategies, it is essential to examine whether and how patient outcomes have changed over the past 40 years in relation to available treatments. This project aims to analyze prognostic trends in MDS, stratified by year of initial diagnosis, to identify associations with specific therapeutic interventions and their potential influence on outcome.

## Patients and methods

The Düsseldorf MDS Registry, established in 1982 is a multicenter registry that systematically collects clinical data on patients with myelodysplastic syndromes including central cytomorphology of blood and bone marrow. Data are contributed by participating investigators and include detailed information on morphology, administered therapies, and clinical outcomes. At the data-collection cut-off date, the registry contained information on 9,128 patients. Data entry is performed by trained personnel using standardized case report forms and does not involve an automated interface with electronic medical records.

We extracted and analyzed data from 1,147 patients diagnosed with myelodysplastic neoplasm (MDS) between 1982 and 2022, for whom we could collect sufficiently detailed information on administered treatment. Inclusion was based solely on the availability of a complete and comprehensible record of all administered therapies. The focus was set specifically on high-risk patients and their recommended therapies. The median age at diagnosis was 65 years (range 18–92). Patients were classified according to the diagnostic information available at the time of their initial assessment. According to the WHO 2022 classification, the majority of patients were diagnosed with MDS-LB (20.2%), MDS-IB-1 (21.5%), or MDS-IB-2 (28.2%). Revised International Prognostic Scoring System (IPSS-R) scores at the time of diagnosis were available for 784 patients; among these, 32.6% were classified as high-risk or very high-risk.

For the purpose of temporal analysis, the study period was divided into three intervals: prior to 2000, 2001–2010, and 2011–2022. Patients were assigned based on the year of initial diagnosis. The analysis focused on high-risk therapies as defined in current clinical guidelines, including allogeneic hematopoietic stem cell transplantation, induction chemotherapy, hypomethylating agents, and low-dose cytoreductive chemotherapy—particularly cytarabine and hydroxyurea. 2. Follow up duration was defined as the time from initial diagnosis to last contact or death.

Patient data were collected and processed using Microsoft Excel 2013, then imported into IBM SPSS Statistics 28 for statistical analysis. Descriptive statistics including median, mean, range, maximum, and minimum were calculated for relevant variables. Kaplan–Meier survival curves were generated, and differences between groups were assessed using the log-rank test, Breslow test, and Tarone-Ware test. For multivariate analysis, we employed the Cox proportional hazards regression model.

## Results

The baseline characteristics of the patients are shown in Table 1.

**Table 1:** Baseline characteristics

	*n* = 1147
Median age (range)	65 (18–92)
Male	59.8%
Female	40.2%
Date of first diagnosis	*n* = 1147
≤2000	25.1%
2001–2010	33.0%
2011–2022	41.9%
WHO2022	*n* = 1144
MDS-LB	20.2%
MDS-H	2.8%
MDS-F	3.6%
MDS-IB1	21.5%
MDS-IB2	28.2%
MDS-del(5q)	3.9%
MDS-SF3B1	6.3%
MDS-biTP53	1.2%
CMML1	6.8%
CMML2	4.2%
MDS-MPN-RS-T	0.8%
MDS-MPN-NOS	0.4%
IPSS-R	*n* = 784
very low	4.8%
low	22.7%
intermediate	24.7%
high	25.3%
very high	22.4%

**Fig. 1:**
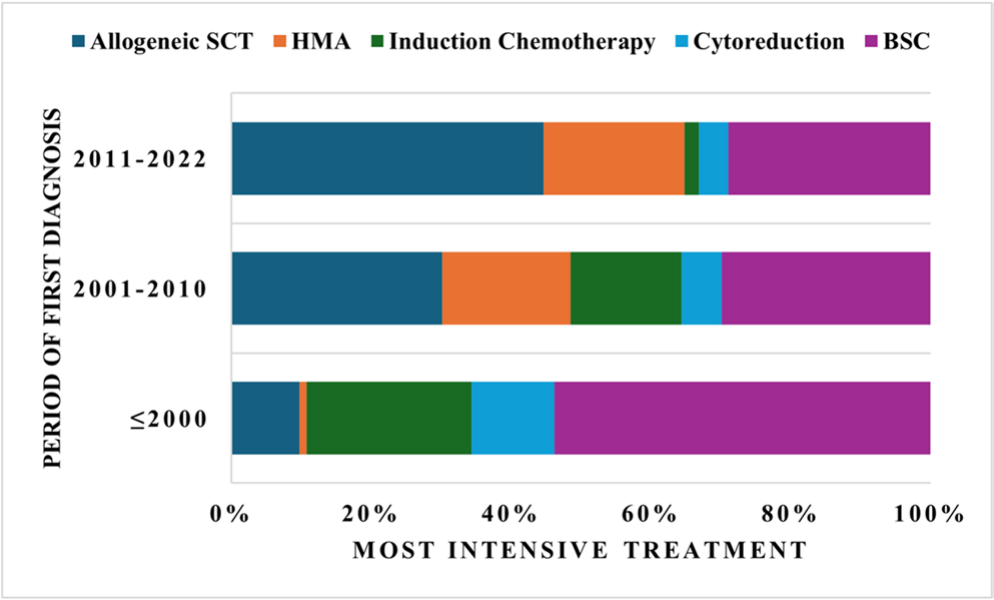
Percentage of patients treated with a specific most intensive treatment grouped by period of first diagnosis. 2011–2022 (44.7%; 20.1%; 2.1%; 4.2%; 28.9%) 2001–2010 (30.2%; 18.3%; 15.9%; 5.7%; 29.9%) ≤ 2000 (9.8%; 1%; 23.6%; 11.8%; 53.8%. *Abb*: SCT = Stem Cell Transplantation, HMA: Hypomethylating agents, BSC: Best supportive care

**Fig. 2:**
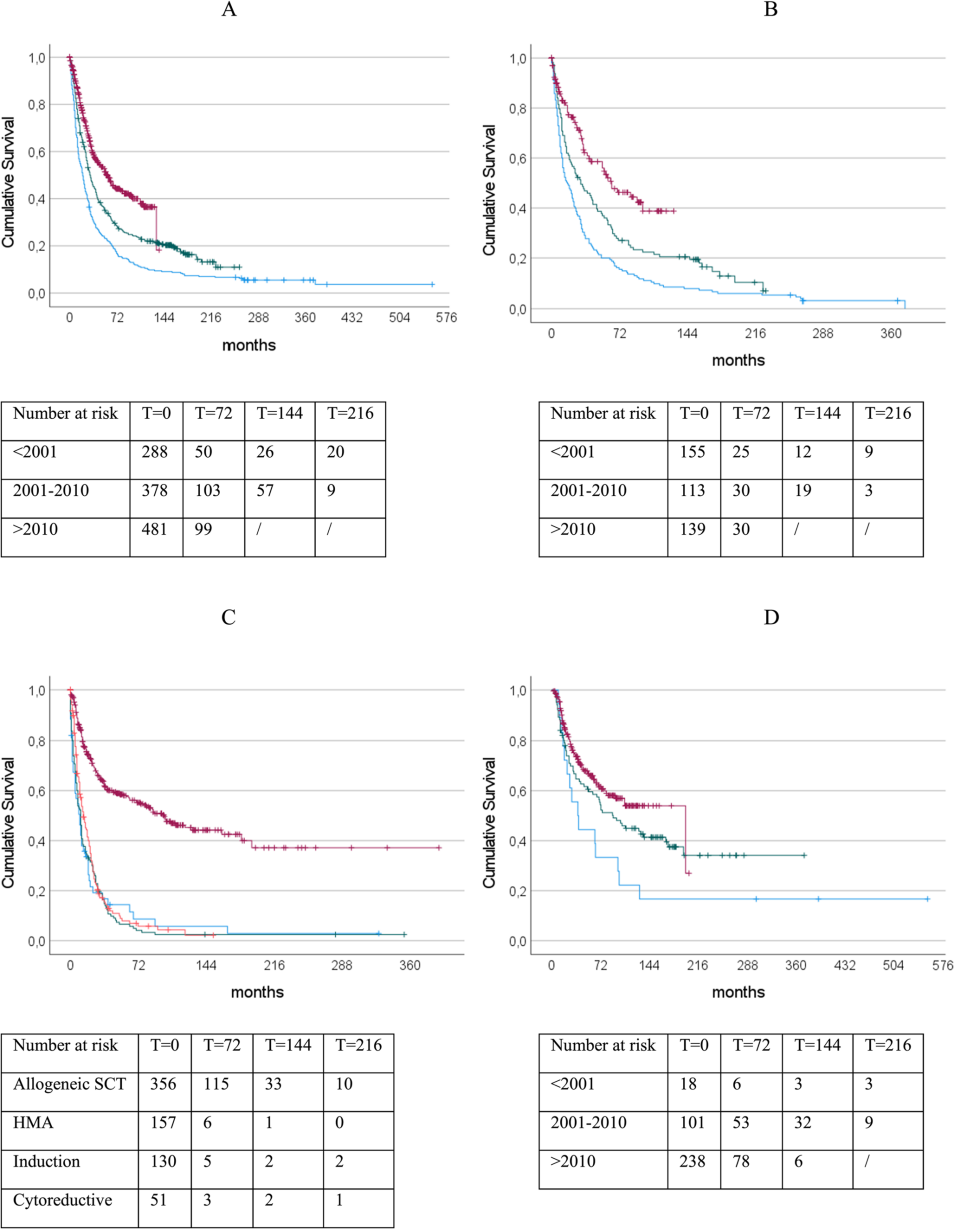
(A) Survival of all patients diagnosed before 2001 (blue, *n* = 288), 2001–2010 (green, *n* = 378) and after 2010 (red, *n* = 481) of 20 months, 31 months and 57 months, respectively (*p* < 0.001). (B) Survival of the patients who received best supportive care (BSC): survival benefit of 64 vs. 31 vs. 17 months in the patient group diagnosed after 2010 (blue, *n* = 155, green, *n* = 113, and red, *n* = 139, *p* < 0.001). (C) Survival of patients since start of treatment compared by most intensive therapy is best in allogeneic transplanted group over the ones who received hypomathylating agents (HMA), Induction chemotherapy and cytoreductive therapy, respectively (100 vs. 14 vs. 11 vs. 10 months, *p* < 0.001; red, *n* = 356, orange, *n* = 157, green, *n* = 130, and blue, *n* = 51). (D) Survival of the allogeneic transplanted patients in the group transplanted before 2001 (blue, *n* = 18), between 2001–2010 (green, *n* = 101) and after 2010 (red, *n* = 238) of 39 months vs. 91 months vs. 198 months, (*p* < 0.001)

The median survival of the entire patient group was 33 months and the median progression time to AML was 167 months, with a total of 35.2% of the patients evolving to AML. In those patients diagnosed prior to 2000 the median survival was 20 months. In the cohort diagnosed between 2001 and 2010, the median survival was 31 months and in the cohort diagnosed between 2011 and 2022 it was with 57 months significant longer (*p* < 0.0001). To investigate this observed improvement, we also analyzed the therapeutic regimens administered to the patients. Overall, 64.5% of the cohort was treated with a high-risk regimen and a total of 31.1% underwent allogeneic stem cell transplantation. An overview of the administered therapies is presented in Table 2. Changes in treatment proportions among MDS patients are shown in Fig. 1. Median survival by subgroup is illustrated in Fig. 2. According to the administered therapies it was observed that the group which was treated with an allogeneic SCT had the longest median survival with 108 months followed by the BSC-group with 31 months and the HMA-group with 27 months. When comparing different time periods within patients receiving the same therapy, survival improved in the BSC group from 17 months to 64 months among the most recently diagnosed patients. This is also seen in the other subgroups; however, the difference is not significant. We performed a multivariate analysis to assess whether the year of first diagnosis has an independent influence on median survival. Therefore we included variables such as most intensive treatment (allogeneic stem cell transplantation vs. induction vs. HMA vs. cytoreduction), IPSS-R, WHO 2022-classification, age at first diagnosis and period of first diagnosis. Table 3 states out the IPSS-R-Score had the strongest impact on survival (Wald statistic = 113.775). After inclusion of the other four variables, the period of diagnosis still demonstrated an independent effect on outcome. Furthermore, we investigated whether the timing of therapy, before or after a possible progression to AML, had an influence on median survival. This effect was observed for all high-risk therapies except HMAs, for which no significant difference was found. In the allogeneic SCT group, median survival was 132 months in patients transplanted earlier compared with 67 months in those transplanted later. The median survival, when defined as the interval from treatment initiation to death or date of last follow up, respectively, was 100 months in the allogeneic SCT group, markedly longer than the 10–14 months observed in the other subgroups.

**Table 2:** Applied therapies

Therapy	*n* = 1147
HR-Therapy	64.5%
other therapy	18.9%
transfusion/no therapy	16.6%
Most intensive therapy	*n* = 1147
No HR-Therapy	35.5%
Allogeneic SCT	31.1%
Induction	12.0%
HMA	14.8%
Cytoreduction	6.6%
Number of treatment lines	*n* = 1147
0	35.5%
1	37.6%
2	20.8%
3	5.1%
4	1.0%

*HR *High-Risk, *SCT *Stem cell transplantation, *HMA *Hypomethylating Agents

**Table 3:** Multivariate analysis

Parameter	χ2	*p*	HR	CI	
Most intensive therapy: BSC	85.383	< 0.001			
Cytoreduction	2.893	0.089	1.435	0.947	2.174
Induction	8.472	0.004	1.484	1.138	1.936
allogeneic SCT	41.104	< 0.001	0.409	0.311	0.538
HMA	3.533	0.060	1.276	0.990	1.646
Age over 65	8.736	0.003	1.371	1.113	1.690
IPSS-R: very low	113.775	< 0,001			
low	0.339	0.560	1.159	0.706	1.903
intermediate	4.624	0.032	1.707	1.049	2.780
high	15.253	< 0.001	2.639	1.622	4.296
very high	35.275	< 0.001	4.443	2.716	7.268
Period of first diagnosis: <2001	8.651	0.013			
2001–2010	0.585	0.444	1.098	0.864	1.394
2011–2022	2.415	0.120	0.803	0.610	1.059

*HR *hazard ratio, *CI *Confidence-Interval, *BSC *Best supportive Care, *SCT *Stem cell transplantation, *HMA *Hypomethylating agents

### AML progression

Among patients diagnosed before 2000 and between 2001 and 2010, the cumulative risk of AML progression at two and five years was comparable (36.4% vs. 36.1% and 49.3% vs. 49.9%). By contrast, in the most recently diagnosed patients, the cumulative risk of AML progression was significantly reduced to 25.6% and 34.2% at two and five years, respectively. In addition, the most intensive treatment had a significant impact on the median time to AML progression. In both the BSC and allogeneic transplantation groups, the median time was not reached at the time of analysis. In the other treatment groups, median time to progression ranged from 15 to 47 months (*p* < 0.001).

### Allogeneic stem cell transplantation

Among patients who underwent allogeneic stem cell transplantation, stratified by treatment decade, an improvement in median survival was observed. In addition, the median age at transplantation increased over time (36.5 years vs. 52 years vs. 61 years), and the proportion of transplanted patients increased markedly (6.3% vs. 22.1% vs. 40%).

## Discussion

This analysis includes 1,147 patients, the earliest of whom was retrospectively diagnosed in 1982, thereby representing a dataset that spans the entire history of MDS.

Since its introduction, allogeneic SCT has remained the only curative treatment option. Despite a therapy-associated mortality of up to 20% [5], it confers a clear survival advantage [6–8]. Ongoing improvement in patient selection [9, 10], timing of transplantation [11, 12], reduction of relapse-risk [13], and lowering of therapy-associated mortality are expected to further improve survival outcomes in MDS [14].

Over the decades, since the initial description of MDS, median survival has steadily increased. Whereas treatment in the 1980 s was largely limited to transfusions and experimental measures, current practice involves structured, risk-adapted therapy tailored to prognosis and comorbidities [15]. Several factors may contribute to improved survival in patients managed with BSC. In our study, the proportion of high-risk patients among later-diagnosed BSC cases decreased from 41% to 25%, in contrast to an increase in the overall cohort. This may reflect broader outpatient management of low-risk patients, expanding therapeutic options for high-risk disease, and improved supportive measures. Rauhe et al. could show that non-curative treatments, like antianemic medication, can improve overall survival [16]. Further it was demonstrated in other studies that iron chelation in transfusion-dependent patients prolongs event-free survival [17]. Moreover, it is debated whether lenalidomide in MDS with isolated del(5q) improves overall survival, with evidence of benefit particularly in responders [18–20]. It was also observed that improved and more efficient use of supportive treatments, such as antibiotics and antifungals, contributed over time to a reduction in infection-related mortality [21]. Nevertheless, the role of supportive therapy on prolonging median survival remains incompletely understood and requires further research.

The survival benefit of allo-SCT was confirmed, with median survival from start of treatment exceeding 100 months compared to 10–14 months for other treatment modalities. Hypomethylating agents (HMAs), currently the first-line treatment for high-risk patients ineligible for transplantation, achieved a median survival of 14 months, which, although superior to alternative non-transplant approaches, did not confer substantial improvement in long-term outcomes. This aligns with findings by Bernal et al., who reported that azacitidine treatment outside of clinical trial settings does not necessarily translate into survival benefits [22]. Current research therefore emphasizes combination strategies, particularly HMA-based regimens with novel agents [12, 15, 23].

Multivariate Cox regression confirmed that the year of initial diagnosis independently influenced overall survival, even when adjusting for treatment, IPSS-R, WHO 2022 classification, and age at diagnosis. Later periods of diagnosis were associated with a lower hazard ratio and improved survival. This is consistent with earlier reports and multifactorial genesis has been discussed [24]. One possible explanation is an earlier individual timing of the initial diagnosis in the course of the disease in recent decades, although this cannot be definitively assessed in our study. The extent to which earlier diagnosis contributes to survival differences remains uncertain, especially given the prognostic stratification at baseline using IPSS-R.

In summary, consistent with our prior study [21], our analysis demonstrates a steady improvement in MDS prognosis over time, with administered therapy playing a decisive role. Further research is needed to clarify the factors contributing to the increase in median survival over time—beyond improved supportive measures and a growing proportion of patients eligible for allogeneic SCT—and to translate this knowledge into improved management of MDS patients.

## Data Availability

All data used in this study is part of the German MDS registry.

## References

[1] Adès L, Itzykson R, Fenaux P (2014) Myelodysplastic syndromes. Lancet Bd 383(9936):2239–2252 10.1016/S0140-6736(13)61901-7 24656536

[2] Steensma DP (2012) „Historical perspectives on myelodysplastic syndromes. Leuk Res 36(12):1441–1452 22921019 10.1016/j.leukres.2012.08.007

[3] Khoury JD, Solary E, Abla O, Akkari Y, Alaggio R, Apperley JF, Bejar R, Berti E, Busque L, Chan JKC, Chen W, Chen X, Chng W-J, Choi JK, Colmenero I, Couplan SE, Cross NCP, De Jong D, Elghetany TM, Takahashi E, Emile J-F, Ferry J, Fogelstrand L, Fontenay M, Germing U, Gujral S, Haferlach T, Harrison C, Hodge JC, Hu S, Jansen JH, Kanagal-Shamanna R, Kantarjian HM, Kratz CP, Li X-Q, Lim MS, Loeb K, Loghavi S, Marcogliese A, Meshinchi S, Michaels P, Naresh KN, Natkunam Y, Nejati R, Ott G, Padron E, Patel KP, Patkar N, Picarsic J, Platzbecker U, Roberts I, Schuh A, Sewell W, Siebert R, Tembhare P, Tyner J, Verstovsek S, Wang W, Wood B, Xiao W, Yeung C and Hochhaus A (2022) The 5th edition of the world health organization classification of haematolymphoid tumours. Myeloid and Histiocytic/Dendritic Neoplasms. Leukemia 36:1703–1719 10.1038/s41375-022-01613-1 PMC9252913 35732831

[4] Fenaux P, Platzbecker U, Adès L (2020) „How we manage adults with myelodysplastic syndrome. Br J Haeematology 189(6):1016–1027 10.1111/bjh.16206 31568568

[5] Vittayawacharin P, Kongtim P, Ciurea SO (2023) „Allogeneic stem cell transplantation for patients with myelodysplastic syndromes. Am J Hematol 98(2):322–337 36251347 10.1002/ajh.26763

[6] Platzbecker U, Schetelig J, Finke J, Trenschel R, Scott BL, Kobbe G, Schaefer-Eckart K, Bornhäuser M, Itzykson R, Germing U, Beelen D, Ehninger G, Fenaux P, Deeg J, Adès L, German MDS, Study (2012) Allogeneic hematopoietic cell transplantation in patients age 60–70 years with de Novo high-risk myelodysplastic syndrome or secondary acute myelogenous leukemia: comparison with patients lacking donors who received azacitidine. Cooperative transplant study groupfred hutchinson cancer research center und groupe francophone de myelodysplasies. Biol Blood Marrow Transplant 9:1415–1421 10.1016/j.bbmt.2012.05.003 PMC4560484 22579634

[7] Robin M, Porcher R, Adès L, Raffoux E, Michallet M, François S, Cahn JY, Delmer A, Wattel E, Vigouroux S, Bay JO, Cornillon J, Huynh A, Nguyen S, Rubio MT, Vincent L, Maillard N, Charbonnier A, de Latour RP, Reman O, Dombret H, Fenaux P, Socié G (2015) HLA-matched allogeneic stem cell transplantation improves outcome of higher risk myelodysplastic syndrome a prospective study on behalf of sfgm-tc and GFM. Leukemia 7:1496–1501 10.1038/leu.2015.37 25676424

[8] Nakamura R, Saber W, Martens MJ, Ramirez A, Scott B, Oran B, Leifer E, Tamari R, Mishra A, Maziarz RT, McGuirk J, Westervelt P, Vasu S, Patnaik M, Kamble R, Forman SJ, Sekeres MA, Appelbaum F, Medizabal A, Logan B, Horowitz M, Cutler C (2021) „Biologic assignment trial of Reduced-Intensity hematopoietic cell transplantation based on donor availability in patients 50–75 years of age with advanced myelodysplastic syndrome. J Clin Oncology: Official J Am Soc Clin Oncol 30:3328–3339 10.1200/JCO.20.03380 PMC8791814 34106753

[9] Oran B (2015) Which patients should undergo allogeneic stem cell transplantation for myelodysplastic syndromes, and when should we do it? Clin Lymphoma Myeloma Leuk 15:43–49 10.1016/j.clml.2015.04.002 26297277

[10] Kröger N, Sockel K, Wolschke C, Bethge W, Schlenk RF, Wolf D, Stadler M, Kobbe G, Wulf G, Bug G, Schäfer-Eckart K, Scheid C, Nolte F, Krönke J, Stelljes M, Beelen D, Heinzelmann M, Haase D, Buchner H, Bleckert G, Giagounidis A (2021) Platzbecker, „Comparison between 5-Azacytidine treatment and allogeneic Stem-Cell transplantation in elderly patients with advanced MDS according to donor availability (VidazaAllo Study). J Clin Oncology: Official J Am Soc Clin Oncol 30:3318–3327 10.1200/JCO.20.02724 34283629

[11] Cutler CS, Lee SJ, Greenberg P, Deeg J, Perez WS, Anasetti C, Bolwell BJ, Cairo MS, Gale RP, Klein JP, Lazarus HM, Liesveld JL, McCarthy PL, Milone GA, Rizzo DJ, Schultz KR, Trigg ME, Keating A, Weisdorf DJ, Antin JH, Horowitz MM (2004) Adecision analysis of allogeneic bone marrow transplantation for the myelodysplastic syndromes: delayed transplantation for low-risk myelodysplasia is associated with improved outcome. Blood Bd 2(104):579–585 10.1182/blood-2004-01-0338 15039286

[12] Oliansky DM, Antin JH, Bennett JM, Deeg J, Engelhard C, Heptinstall KV, de Lima M, Gore SD, Potts RG, Silverman LR, Jonas RB, McCarthy PL, Hahn T (2009) „The role of cytotoxic therapy with hematopoietic stem cell transplantation in the therapy of myelodysplastic syndromes: an evidence-based review. Biology Blood Marrow Transplantation: J Am Soc Blood Marrow Transplantation 2:137–172 10.1016/j.bbmt.2008.12.003 19167676

[13] Phelan R, Chen M, Bupp C, Bolon YT, Broglie L, Brunner-Grady J, Burns LJ, Chhabra S, Christianson D, Cusatis R, Devine SM, D’Souza A, Eapen M, Hamadani M, Hengen M, Lee SJ, Moskop A, Page KM, Pasquini M, Pérez WS, Riches M, Rizzo D, Saber W, Spellman SR, Stefanski HE, Steinert P, Weisdorf D, Horowitz M, Auletta JJ, Shaw BE, Arora M (2002) Updated trends in hematopoietic cell transplantation in the United States with an additional focus on adolescent and young adult transplantation activity and outcomes. Transplant Cell Ther 7:409 10.1016/j.jtct.2022.04.012 PMC9840526 35447374

[14] Kobbe G, Schroeder T, Haas R (2018) Germing, „The current and future role of stem cells in myelodysplastic syndrome therapies. Expert Rev Hematol 5(11):411–422 10.1080/17474086.2018.1452611 29547016

[15] Platzbecker U (2019) Treatment of MDS. Blood Bd 133(10):1096–1107 10.1182/blood-2018-10-844696 30670446

[16] Rauhe K, Kasprzak A, Schulz F, Nachtkamp K, Strupp C, Kündgen A, Dietrich S, Mayer K, Götze KS, Hofmann W-K, Giagounidis A, Gattermann N, Germing U (2025) Non-curative therapies and their impact on the prognosis of patients with myelodysplastic syndromes- a retrospective matched-pairs analysis. Ann Hematol 6:3281–3288 10.1007/s00277-025-06485-w PMC12283770 40576688

[17] Angelucci E, Li J, Greenberg P, Wu D, Hou M, Horacio Monato Figueroa E, Guadalupe Rodriguez M, Dong X, Ghosh J, Izquierdo M, Garcia-Manero G (2020) Iron chelation in transfusion-dependent patients with low- to intermediate-1-risk myelodysplastic syndromes: a randomized trial. Ann Intern Med 172(8):513–522 32203980 10.7326/M19-0916

[18] Garcia-Manero G (2023) „Myelodysplastic syndromes: 2023 update on diagnosis, risk-stratification and management. Am J Hematol 98:1307–1325 37288607 10.1002/ajh.26984 PMC12002404

[19] Fenaux P, Giagounidis A, Selleslag D, Beyne-Rauzy O, Mufti G, Mittelman M, Muus P, te Boekhorst P, Sanz G, Cdel Cañizo, Guerci-Bresler A, Nilsson L, Platzbecker U, Lübbert M, Quesnel B, Cazzola M, Ganser A, Bowen D, Schlegelberger B, Aul C, Hellström-Lindberg E (2011) A randomized phase 3 study of lenalidomide versus placebo in rbc transfusion-dependent patients with low-/intermediate-1-risk myelodysplastic syndromes with del5q. Blood Bd 14(118):3765–3776 10.1182/blood-2011-01-330126 21753188

[20] Giagounidis A, Mufti GJ, Mittelman M, Sanz G, Platzbecker U, Muus P, Selleslag D, Beyne-Rauzy O, te Boekhorst P, del Cañizo C, Guerci-Bresler A, Nilsson L, Lübbert M, Quesnel B, Ganser A, Bowen D, Schlegelberger B, Göhring G, Fu T, Benettaib B, Hellström-Lindberg E (2014) Fenaux, „Outcomes in RBC transfusion-dependent patients with Low-/Intermediate-1-risk myelodysplastic syndromes with isolated deletion 5q treated with lenalidomide: a subset analysis from the MDS-004 study. Eur J Haematol 5:429–438 10.1111/ejh.12380 PMC4232868 24813620

[21] Neukirchen J, Lauseker M, Blum S, Giagounidis A, Lübbert M, Martino S, Siragusa S, Schlenk RF, Platzbecker U, Hofmann W-K, Götze K, Palumbo GA, Magrin S, Kündgen A, Aul C, Hildebrandt B, Hasford J, Kobbe G, Haas R (2014) Germing, „Validation of the revised international prognostic scoring system (IPSS-R) in patients with myelodysplastic syndrome: A multicenter study. Leuk Res 38(1):57–64 24238640 10.1016/j.leukres.2013.10.013

[22] Bernal T, Martinez-Camblor P, Sanchez-Garcia J, de Paz R, Luño E, Nomdedeu B, Ardanaz MT, Pedro C, Amigo ML, Xicoy B, del Cañizo C, Tormo M, Bargay J, Valcarcel D, Brunet S, Benlloch L, Sanz G (2015) Effectiveness of azacitidine in unselected high-risk myelodysplastic syndromes: results from the Spanish registry. Leukemia Bd 9(29):1875–1881 10.1038/leu.2015.115 25943181

[23] Adès L (2023) Traitement des syndromes myélodysplasiques de haut risque. Bulletin du Cancer 11:1162–1167 10.1016/j.bulcan.2023.02.029 37407322

[24] Neukirchen J, Nachtkamp K, Schemenau J, Aul C, Giagounidis A, Strupp C, Kuendgen A, Kobbe G, Haas R, Germing U (2015) „Change of prognosis of patients with myelodysplastic syndromes during the last 30 years. Leuk Res 39(7):679–683 25929166 10.1016/j.leukres.2015.04.001

